# Nasopharyngeal Carcinoma With Bilateral Breast Metastasis: A Report of an Elusive Case

**DOI:** 10.7759/cureus.27497

**Published:** 2022-07-31

**Authors:** Sanjay Vikram Soni, Vikas Malhotra, Raman Sharma, Meeta Singh, Rashmi Dixit, Varuna Mallya

**Affiliations:** 1 Otolaryngology - Head and Neck Surgery, Maulana Azad Medical College, New Delhi, IND

**Keywords:** cancer, tumor, nasopharyngeal, metastasis, carcinoma, breast

## Abstract

Nasopharyngeal carcinoma is a prevalent head and neck cancer, especially in Southeast Asia. Although its potential for distant metastasis is well established, metastasis to the breast has seldom been reported. To the best of our knowledge, this is the fourth report of a case of nasopharyngeal carcinoma metastasizing to bilateral breasts. A 35-year-old patient presented with left nasal obstruction, epistaxis, and a palpable mass in her left breast, without any cervical or axillary lymph nodal enlargement. Radiological examination with contrast-enhanced computed tomography scan and magnetic resonance and imaging of breast revealed the presence of enhancing mass lesions in bilateral breasts. Histopathology of the nasal mass was suggestive of undifferentiated nasopharyngeal carcinoma. Breast fine needle aspiration revealed an abundance of metastatic squamous cells. Immunohistochemistry examination was positive for chromogranin A, synaptophysin, and cluster of differentiation-56, confirming the diagnosis of a primary nasopharyngeal malignancy metastasizing to bilateral breasts. Differentiation between metastatic disease and a coexisting second primary is imperative for planning appropriate treatment and defining the further outcomes.

## Introduction

The nasopharynx is a tubular formation that connects the nose to the oropharynx. The most common site for the origin of nasopharyngeal carcinoma (NPC), as described in the literature, is the fossa of Rosenmuller (FOR), also known as the pharyngeal recess. In most of the world, NPC is a rare tumor. However, in Southeast Asia, it comprises 18% of all malignant tumors [1]. NPC classically shows the bimodal age distribution of 16-20 and 46-50 years, with a slight male preponderance [2]. Recent data reveals India has a low incidence (age-standardized rate <1/100000 males) of the disease, with the male-to-female ratio being 2-3:1 [3]. Multiple etiological factors are found to be associated with the condition. These include genetics, smoking, high titers of Epstein-Barr virus, a diet consisting of preserved foods containing nitrosamines, and a family history of nasopharyngeal cancer.

Usual manifestations of NPC include nasal bleeding and obstruction, and painless neck lump since cervical lymph node metastasis occurs early in the course of the disease. Other common manifestations include aural fullness and hearing loss due to Eustachian tube blockage. It is unusual to have ophthalmological presentation as the initial feature [2]. NPC has a greater incidence of distant metastasis than other head and neck tumors.

The most common site of hematogenous metastasis is the axial skeleton, while that of lymphatic metastasis is cervical or retropharyngeal lymph nodes [4]. At the time of primary diagnosis, approximately one-third of patients present with disseminated disease [5]. Metastasis to the breast, however, is extremely rare and usually associated with disseminated disease. Overall, the most common primaries to metastasize to the breast in the decreasing order of incidence include the contralateral breast, melanoma, lymphoma, lung cancer, and ovarian cancer [6,7].

A comprehensive search of English medical literature divulged very few reports of a primary carcinoma of nasopharynx metastasizing to the breast, even fewer to bilateral breasts [1-7]. There have been only 12 previously reported cases of NPC metastasizing to the breast, and only three case reports have reported it in bilateral breasts [8-10]. The present case study describes the 13th case overall and the fourth case of NPC metastasizing to bilateral breasts.

## Case presentation

**Figure 1:**
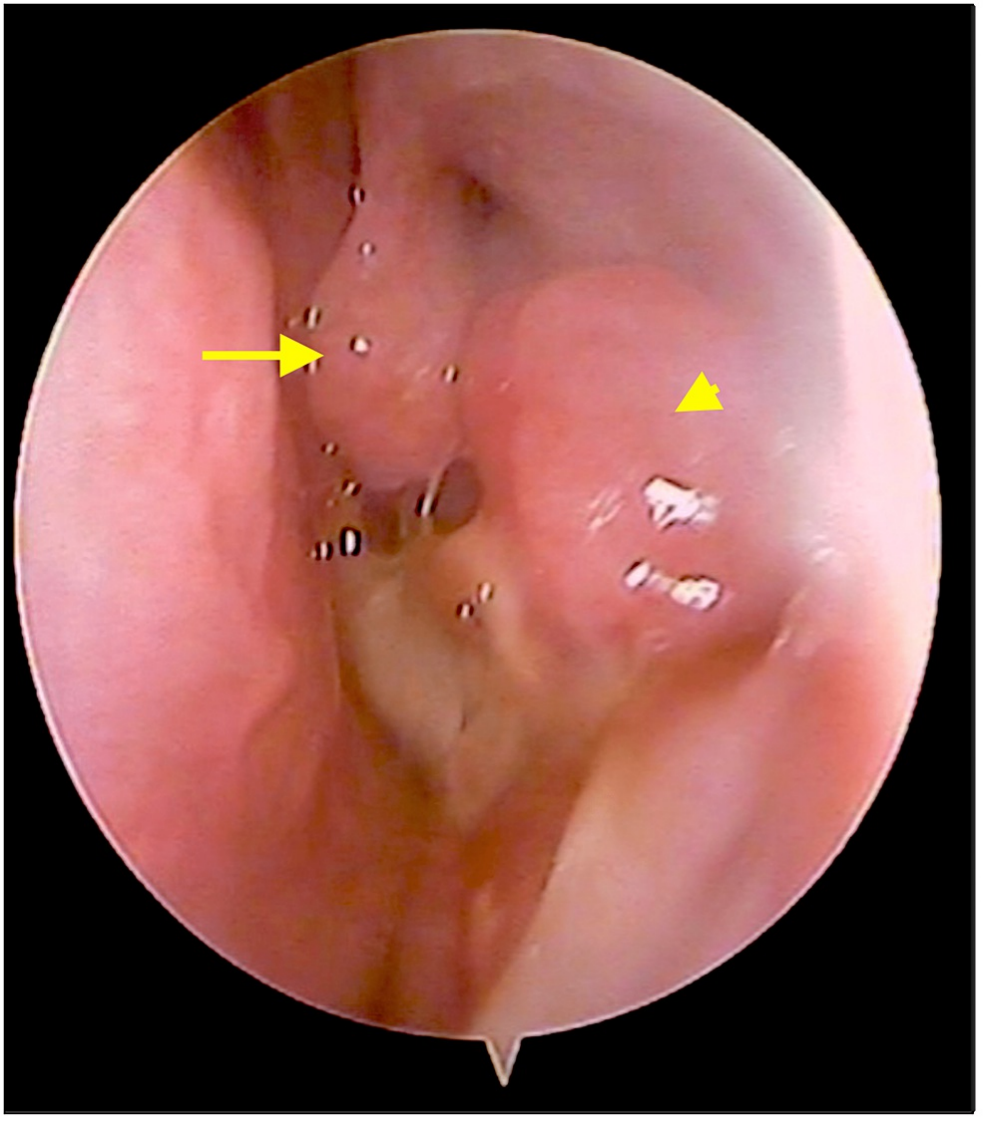
Endoscopic view of left nasal cavity. yellow arrow: middle turbinate; yellow arrowhead: tumor mass

A 35-year-old lady presented to the outpatient department with complaints of recurrent attacks of left-sided profuse nasal bleed, left-sided nasal obstruction, and left-sided facial anesthesia of one-year duration with an increase in the frequency of episodes of epistaxis and nasal obstruction for one month. Also, there was a history of pathological right clavicle fracture, persistent backache and dull aching skeletal pain, persistent headache, pain abdomen, and melena. There was no history of neck lump, ear fullness, decreased hearing, substance addiction, or relevant family/medical history. Clinical examination revealed a red fleshy mass in the left nasal cavity extending from the nares anteriorly to the choana posteriorly that bled on touch (Figure 1). There was fullness over the left side of the face, and the left nasolabial fold was obliterated, associated with anesthesia over the left side of the face. There was no palpable cervical lymph node.

An ophthalmological assessment revealed ptosis of the left eye with cranial nerve VI and cranial nerve II palsy leading to diplopia and left pupil being non-reactive, respectively. Also, there was a clinically palpable lump in the left breast, measuring 5 x 4 cm, involving the lower outer quadrant and the nipple-areola complex, not fixed to the skin or underlying muscle and without any axillary lymphadenopathy. The right breast, however, did not have any clinically palpable lump.

A contrast-enhanced computed tomography (CECT) scan of the nose and paranasal septum (PNS), chest, and abdomen was undertaken along with a skeletal survey because of high suspicion of skeletal metastasis. In addition, magnetic resonance imaging (MRI) breast was performed to assess the breast lesion.

**Figure 2:**
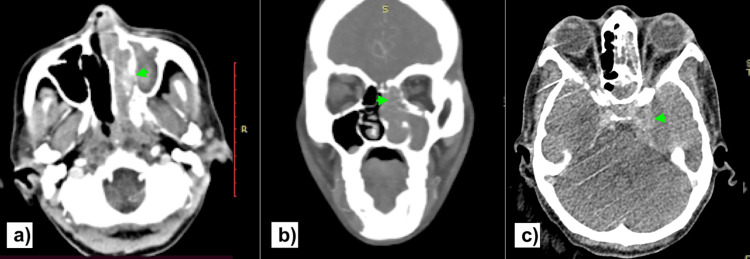
Contrast-enhanced computed tomography of the nose and paranasal sinuses revealed an enhancing soft tissue mass lesion in the nasopharynx extending into the left nasal cavity (a), left maxillary, ethmoid, and frontal sinus (b), and into the middle cranial fossa in the region of the cavernous sinus (c), all marked with green arrowheads.

CECT of the nose and PNS revealed an enhancing soft tissue mass lesion in the nasopharynx extending into the left nasal cavity (Figure 2a), left maxillary, ethmoid, and frontal sinus (Figure 2b), and into the middle cranial fossa in the region of the cavernous sinus (Figure 2c).

CECT of the chest revealed a heterogeneously enhancing lesion (measuring 3.4 x 3.7 x 3.2 cm^3^) in left breast parenchyma with calcifications, and a few well-defined homogenously enhancing lesions in bilateral breast parenchyma, the largest measuring 1.6 x 1.6 x 1.6 cm^3^ in the right breast.

**Figure 3:**
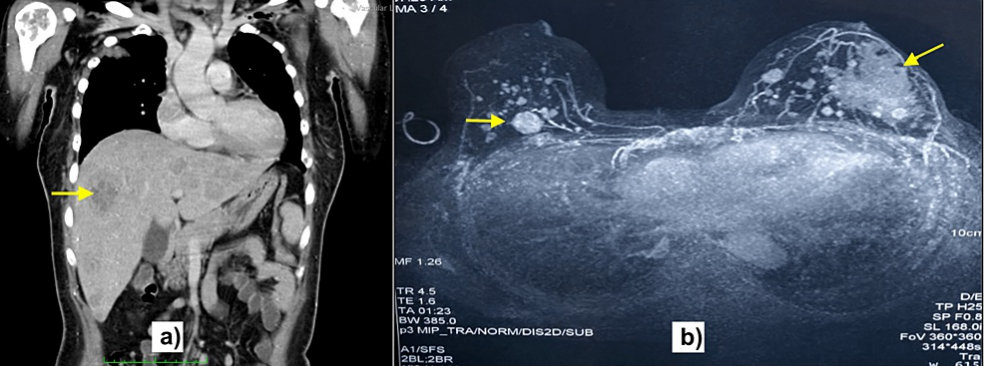
Contrast-enhanced computed tomography abdomen showing targetoid pattern metastasis (yellow arrow) in the liver, b) circumscribed masses scattered in the bilateral breast (yellow arrows) parenchyma with a heterogeneously enhancing mass with irregular margins in the lower outer quadrant of the left breast.

CECT abdomen revealed multiple well-defined, variable-sized rounded lesions involving both the liver lobes with a targetoid pattern of peripheral enhancement, suggestive of metastasis (Figure 3a). Also, there were multiple lytic lesions involving axial and appendicular skeleton- vertebral bodies, sternum, ribs, pelvis, bilateral clavicles, and head of the bilateral humerus.

MRI bilateral breast revealed multiple small round-oval-shaped and circumscribed masses scattered in the bilateral breast parenchyma. Also, there was a heterogeneously enhancing mass with irregular margins in the lower outer quadrant of the left breast (Figure 3b). The bilateral breast scan was reported as breast imaging reporting and data system (BI-RADS) category 5.

**Figure 4:**
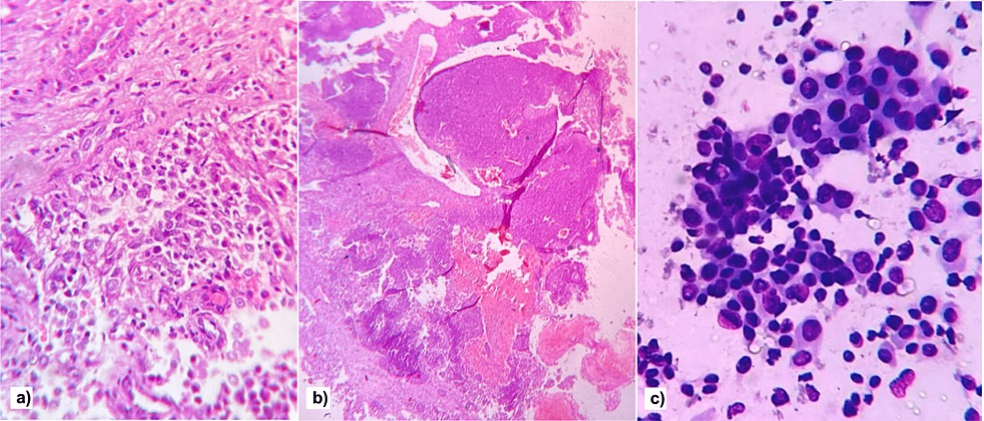
(a) Photomicrograph showing atypical cells having scanty cytoplasm and large nucleus, no keratinization; (b) photomicrograph shows nests of atypical cells, and (c) photomicrograph showing atypical cells in 3D clusters and dissociated in the background having a moderate amount of cytoplasm, hyperchromatic nucleus, nuclear irregularity, and moderate nuclear pleomorphism.

Thus, given the clinical-radiological findings, in addition to a sinonasal primary tumor, possibilities of primary breast malignancy and multiple myeloma were also considered, and histopathology and cytopathology assistance was employed to reach the final diagnosis. Histopathology of tissue from the left nasal cavity revealed a non-keratinizing squamous cell carcinoma (undifferentiated type) of the nasopharynx (Figure 4).

Ultrasound-guided fine-needle aspiration was then performed from the breasts and the liver lesions. The cytopathology favored the diagnosis of metastatic disease to bilateral breasts and liver, similar to NPC (Figure 4). In addition, immunohistological examination revealed cells positive for chromogranin A, synaptophysin, and CD 56. These markers are not expressed routinely in normal breast epithelial cells.

In the hematological profile, the patient was anemic and had markedly raised serum alkaline phosphatase (ALP). However, no M-band was seen on serum electrophoresis. Urine for Bence-Jones proteins was negative. Kidney function tests and serum calcium were normal. Also, an extensive medical literature search revealed no reported cases of primary NPC occurring simultaneously with multiple myeloma, except for a rare case of a patient with multiple myeloma on chemotherapy with lenalidomide who developed NPC [11]. Chemotherapy is the first-line treatment if multiple metastases are diagnosed. Hence, the patient was referred for radio-chemotherapy for further management. The patient received docetaxel plus 5-fluorouracil and cisplatin-based chemotherapy for four cycles and has been asymptomatic for the past eight months after treatment initiation.

## Discussion

NPC can remain insidious when the primary tumor is relatively small; hence, delay in seeking professional treatment is common. If NPC is clinically suspected, nasal endoscopy and biopsy of the FOR are recommended even when no obvious growth is seen. The presentation usually depends on the extent of the disease.

It is difficult to diagnose a breast lump as a metastatic disease from NPC on clinical grounds alone. Careful corroboration with radiological and histopathological evidence is extremely important to clinch the diagnosis. An unfortunate misdiagnosis as primary carcinoma breast could lead to unnecessary mastectomy while the primary disease may progress unchecked. Two cases of NPC metastasizing to the breast in males also have been reported [12]. Metastasis to either uni-or-bilateral breasts changes the treatment plan to palliative chemo-radiation. In the rare occurrence of a synchronous breast and nasopharyngeal malignancy, the management stands individualized and depends on multiple factors. These include the statuses of estrogen receptor (ER), progesterone receptor (PR), and human epidermal growth factor receptor 2 (HER2), the local stage of the breast lesion, and paclitaxel/carboplatin-based chemotherapy for disseminated NPC. Compared to primary breast malignancy, metastases to the breasts are more likely to be superficial, less likely to be fixed to the underlying muscle or skin, and round and well marginated [8]. Additionally, In the setting of a histopathologically proven primary in the nasopharynx, various tests were carried out to ascertain the nature of the co-existing breast lump. These included MRI breast, USG-guided fine needle aspiration from both the breasts, and Immunohistochemistry. The cytology findings were consistent with atypical squamous cells which resembled the primary in the nasopharynx.

At the time of presentation, approximately one-third of the patients with NPC have distant metastasis [13]. The presence of elevated ALP levels correlates with the involvement of more than one metastatic site and skeletal metastasis. The skeleton is the most common site of distant metastasis for NPC, and most bony metastases are osteolytic. The red marrow containing proximal long bones and axial skeleton are preferentially involved as most skeletal metastases are hematogenous [14]. CT scans are the preferred modality for detecting skeletal metastasis since radiographs have low sensitivity (sensitive only for metastatic lesions > 1 cm diameter).

Anemia is a powerful contributory factor to tumor growth and dissemination. In an anemic state, the oxygen transport capacity of blood is lowered, further reducing the oxygen supply to the tumor. In response, tumor hypoxia leads to the release of apoptotic, proliferative, and angiogenic signals. In addition, this persistent hypoxic state in tumors can be a barrier against systemic chemotherapeutic agents [15]. 

World health organization (WHO) has classified NPC into three groups: non-keratinizing carcinoma (differentiated or undifferentiated), keratinizing squamous cell carcinoma, and basaloid squamous cell carcinoma. The non-keratinizing undifferentiated is the commonest form.

Cranial nerve paralysis (leading to ophthalmic involvement) is an uncommon presentation of NPC, with frequency ranging from 13-29% [16]. The most commonly involved cranial nerves are V, VI, IX, and X, accounting for 50% of all palsies [3]. In addition, orbital involvement from NPC is reported in 3.2% of cases [17].

NPC is a radiosensitive tumor, the primary treatment modality being external beam radiotherapy. The usual radiation dose of 66 to 70 GY is delivered to the nasopharynx. This dose is usually given a 1.8 to 2.0 GY per day fraction through two lateral opposing fields with or without an anterior field [18]. However, the outcome in such patients remains poor owing to multiple distant metastases. Paclitaxel/docetaxel and cisplatin-based chemotherapy is considered for disseminated disease [19].

## Conclusions

The NPC, although rare, can present with extensive metastasis. A high index of suspicion for such malignancy in patients with nasopharyngeal obstructive mass should be considered. A prompt endoscopic and radiological workup can help in the timely detection of this malignancy, considering that established metastatic disease has implications on both the preferred treatment modality and prognosis. Docetaxel plus 5-fluorouracil and cisplatin-based chemotherapy is an effective treatment modality in the management of such cases. Although infrequent, metastasis to the breast from NPC should be contemplated and ruled out by a thorough clinical examination and relevant radiological investigations, especially in cases of advanced disease. An unfortunate misdiagnosis as primary carcinoma breast could lead to unnecessary mastectomy or a hormone receptor status-driven chemotherapy while the primary disease (NPC) may progress unchecked.
